# *IFI35* and *IFIT3* are potentially important biomarkers for early diagnosis and treatment of esophageal squamous cell carcinoma: based on WGCNA and machine learning analysis

**DOI:** 10.3389/fgene.2025.1583202

**Published:** 2025-05-20

**Authors:** Hao Wu, Liang Yang, Xiaokun Weng

**Affiliations:** ^1^ First School of Clinical Medicine, Gansu University of Chinese Medicine, Lanzhou, Gansu, China; ^2^ Department of Neurosurgery, Shanghai Jiao Tong University Affiliated Sixth People’s Hospital South Campus, Shanghai, China; ^3^ Department of Radiotherapy, Lishui People’s Hospital, Lishui, Zhejiang, China

**Keywords:** ESCC, IFIT3, IFI35, WGCNA, machine learning

## Abstract

**Background:**

Esophageal squamous cell carcinoma (ESCC) does not have distinct and highly sensitive biomarkers, making its diagnosis difficult. Consequently, identifying dependable biomarkers is critical, as these indicators can facilitate accurate ESCC diagnosis and enable effective prognostic evaluation.

**Methods:**

ESCC datasets (GSE29001, GSE20347, GSE45670, and GSE161533) were sourced from the GEO, and the Limma package identified differentially expressed genes (DEGs). To characterize co-expression network, weighted gene co-expression network analysis (WGCNA) was performed, allowing for the identification of relevant co-expression modules. To assess the biological pathways of intersecting genes, we performed pathway enrichment analysis using Kyoto Encyclopedia of Genes and Genomes (KEGG) and Gene Ontology (GO). The Support Vector Machine Recursive Feature Elimination (SVM), along with Least Absolute Shrinkage and Selection Operator (LASSO) regression, was applied to identify clinical biomarkers. Finally, the differences of immune cell infiltration were also detected.

**Results:**

1,019 genes were derived by integrating DEGs with co-expressed module genes. KEGG and GO revealed a strong association between these genes and processes such as chemotaxis and IL−17 signaling pathways. Two hub genes (*IFIT3* and *IFI35*) were selected through LASSO regression and SVM. Additionally, ROC curve analysis confirmed their potential for reliable diagnostic performance. Furthermore, differences in immune cell infiltration were observed.

**Conclusion:**

Collectively, *IFIT3* and *IFI35* emerged as promising candidate biomarkers, offering novel insights to enhance early detection and guide targeted treatment strategies for ESCC.

## 1 Introduction

Ranked as the eighth most prevalent malignancy globally, esophageal cancer comprises approximately 3% of all cancer diagnoses. Despite its moderate incidence, the disease is associated with unfavorable outcomes and stands as the sixth leading contributor to cancer mortality worldwide (Morgan et al., 2022). Esophageal cancer comprises two primary histological subtypes: squamous cell carcinoma, which accounts for 85% of cases, and adenocarcinoma, representing the remaining 14%, with squamous cell carcinoma accounting for 90% of cases in China (Chen, 2015; Yang et al., 2024; Zhao et al., 2024). Most patients are diagnosed during late-stage disease progression and have lost the opportunity for surgery, being limited to radiotherapy, chemotherapy, or immunotherapy. Although significant progress has been made in therapeutic modalities over recent decades, no breakthrough in the efficacy of esophageal cancer treatment has been achieved, and the 5-year survival rate remains low (Cheng et al., 2024; Zou et al., 2024). The absence of well-characterized highly sensitive biomarkers for ESCC poses significant challenges to its precise diagnosis and effective clinical management. This critical gap in biomarker identification not only impedes early disease detection but also complicates the development of targeted therapeutic strategies, ultimately limiting opportunities for personalized interventions. Therefore, discovering robust biomarkers is essential to enhance diagnostic precision and improve prognostic stratification in ESCC.

Recent advances in bioinformatics have enabled comprehensive analysis of genes associated with ESCC. In the study, sequencing data from publicly available repositories were analyzed, and the WGCNA algorithm was employed to identify candidate genes clusters with high correlations. These clusters were then associated with modular trait genes or central genes within the modules, and the module membership index was calculated (Cui et al., 2024). We subsequently intersected the selected differentially expressed genes and conducted KEGG and GO analyses to identify shared pathogenic mechanisms. The hub genes were pinpointed by LASSO and SVM, followed by an evaluation of their predictive capabilities. Furthermore, we examined immune cell infiltration to compare the differences between normal esophageal epithelium and esophageal cancer.

## 2 Materials and methods

### 2.1 Data source and preprocessing

Based on the selection criteria outlined in previous studies (Alotaibi et al., 2023; Sun et al., 2021), we identified 4 datasets: GSE29001, GSE20347, GSE45670, and GSE161533. We selected the GSE29001 dataset and performed GeneSymbol mapping. Log2 (x+1) transformation was applied to the data, and “normalizeBetweenArrays” function was conducted to correct for batch effects. We subsequently employed the “limma” to select DEGs (Ritchie et al., 2015). The threshold for DEG identification was set at P < 0.05. We then identified 4,122 differentially expressed genes.

### 2.2 WGCNA analysis and module identification

For the WGCNA, the input matrix was generated using all genes from the GSE29001 dataset. A soft threshold of 1–20 was used for topology calculation and then the optimal soft threshold was further selected. Adjacency matrices were subsequently computed and converted into a Topological Overlap Matrix (TOM) to evaluate network interconnectedness. The hierarchical cluster tree was constructed by calculating the differential extent. Modules exhibiting similar expressions were identified and combined. Clinically relevant modules exhibiting significant correlations with disease traits were prioritized for subsequent functional exploration. Then, we use module membership (MM) and Gene significance (GS) for further analyzing (Sun et al., 2024).

### 2.3 Enrichment analysis

Functional annotation of candidate genes was conducted through GO and KEGG pathway analyses, implemented via the clusterProfiler toolkit in R (Yu et al., 2012). In addition, the results of GO analysis were also obtained by metscape.

### 2.4 PPI networks construction

The selected genes were input into the STRING database (https://string-db.org/), and then Cytoscape was used to construct a network. The top 10 genes in the PPI network were identified using the MCC algorithm in Cytoscape.

### 2.5 Machine learning approach for selecting diagnostic biomarkers

LASSO and SVM (Xie et al., 2023; Valkenborg et al., 2023) were used as machine learning approaches. SVM was used to pinpoint hub genes by filtering redundant features from high-dimensional datasets. And the LASSO model was built to identify critical genes. By using the MCC method the top 10 genes were chosen, followed by further biomarker screening with SVM and LASSO.

### 2.6 Evaluating immune cell infiltration

To systematically characterize the immune microenvironment, a computational deconvolution approach (CIBERSORT) was employed to quantify the relative abundance of distinct lymphocyte subsets through analysis of cell type-specific gene signatures. The analytical framework integrated infiltration patterns across 22 functionally annotated immune cell populations, ultimately constructing a comprehensive immune infiltration matrix for subsequent multidimensional evaluation. In parallel, non-parametric correlation analysis utilizing Spearman’s rank-order coefficients was implemented to explore potential connections of immune cell infiltration dynamics and critical biomarkers (Newman et al., 2015).

### 2.7 Identification of potential drugs

The candidate genes of ESCC were input into the Enrichr platform (https://maayanlab.cloud/Enrichr/) (Chen et al., 2013). We then employed Drug Signatures Database (DSigDB) to select the potential drugs (Yoo et al., 2015).

### 2.8 Statistical analysis

Data analysis was performed using R4.4.1. A T-test was used to compare normally distributed data, while the Wilcoxon test was used to compare nonnormally distributed data between the control group and the tumor group. P < 0.05 was considered statistically significant.

## 3 Results

### 3.1 Identification of DEGs

**FIGURE 1:**
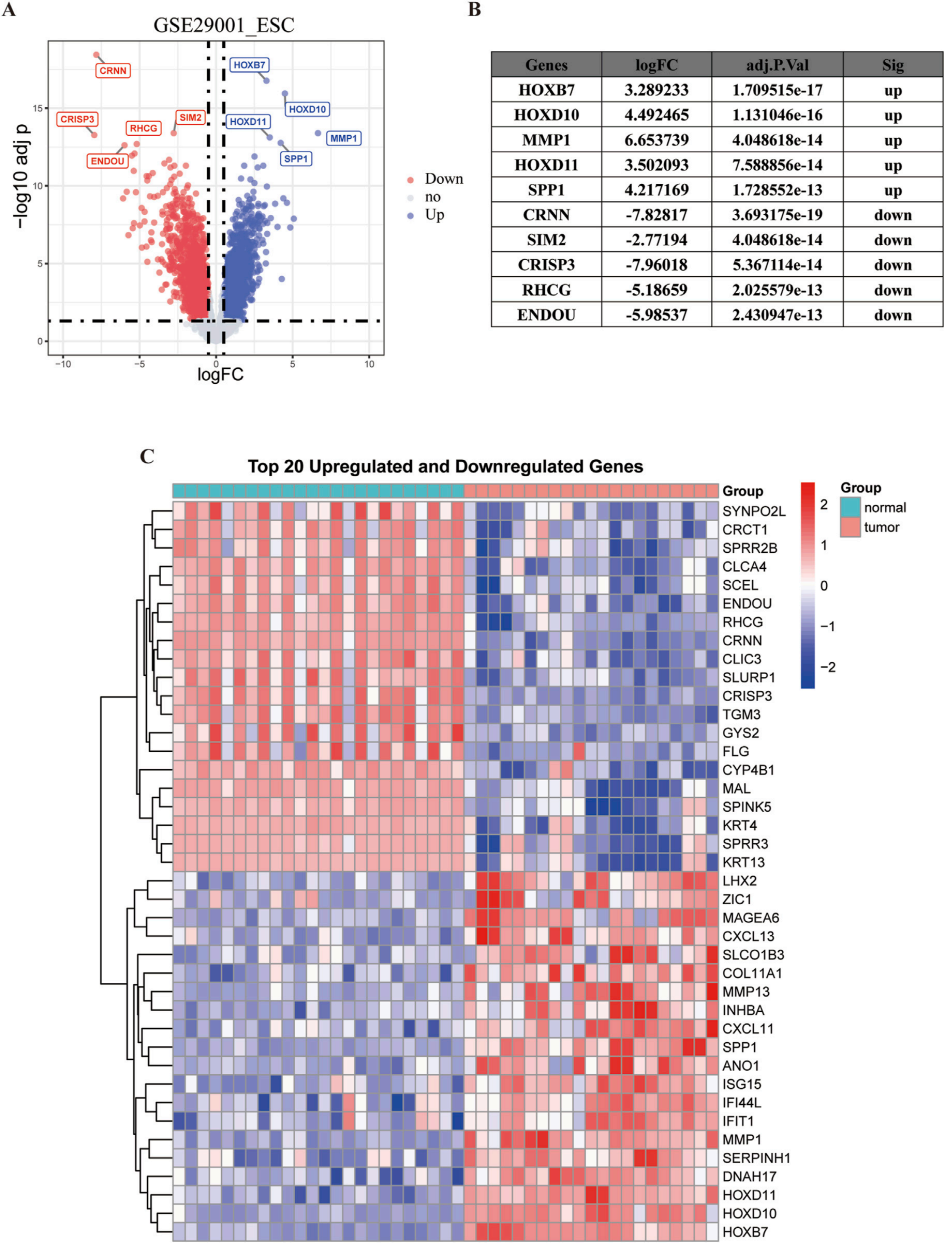
Visualization of differentially expressed genes. The red dots represent genes that are significantly downregulated and the blue dots represent significantly upregulated genes. **(A)** Volcano plot. **(B)** Top 5 upregulated and downregulated genes with the most significant differences. **(C)** Heatmap of the first 20 upregulated genes and 20 downregulated genes in GSE29001.

Analysis of the GSE29001 dataset revealed 4,122 genes exhibiting differential expression, as shown in a volcano plot displaying 1980 downregulated and 2,142 upregulated genes. And a figure was provided to show the five most significantly downregulated and upregulated genes (Figures 1A,B). In addition, a heatmap shows the top 20 most significantly downregulated and upregulated genes (Figure 1C).

### 3.2 WGCNA of ESCC

**FIGURE 2:**
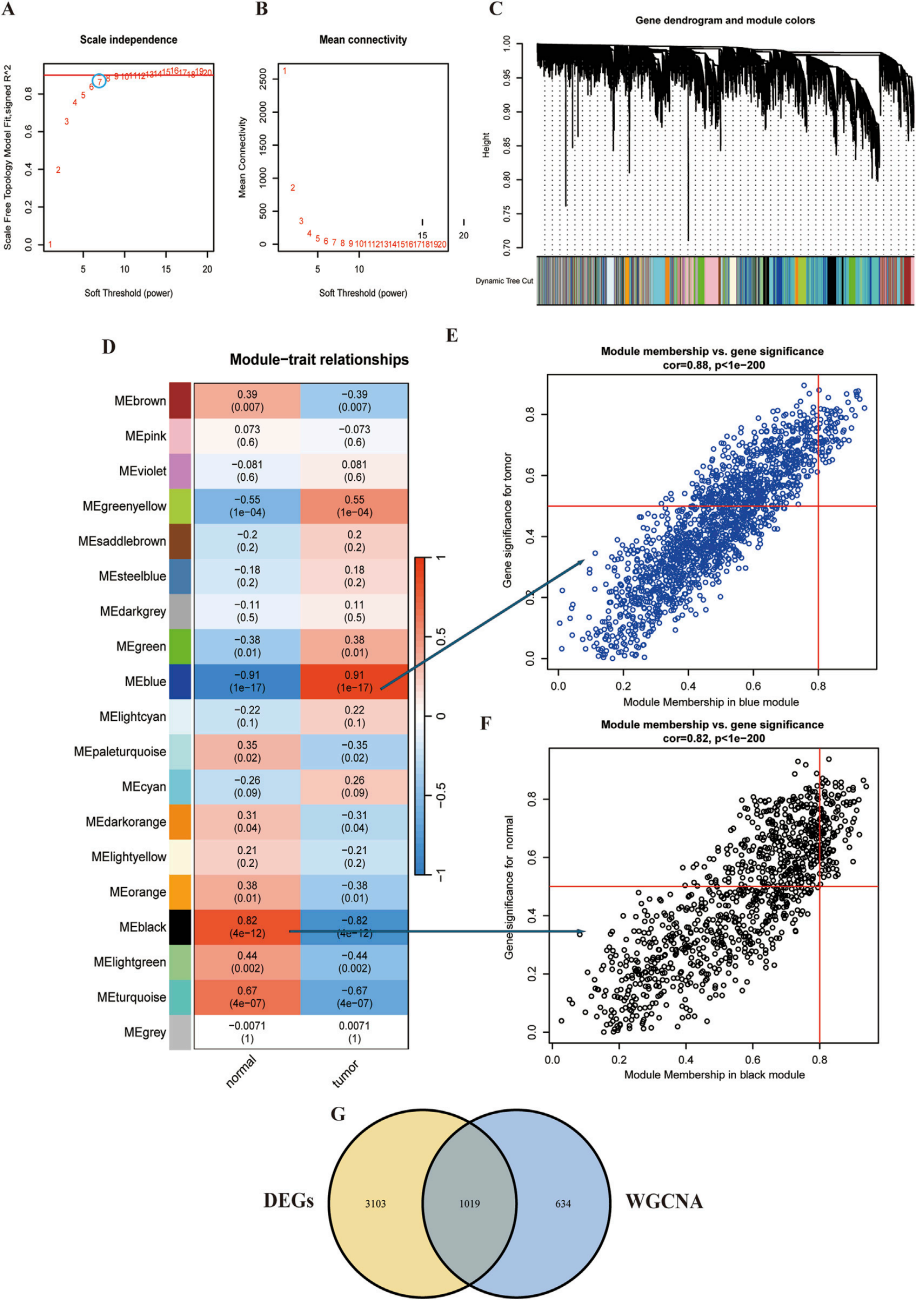
WCGNA of ESCC. **(A,B)** Mean connectivity for scale independence and soft threshold (β) in the GSE29001 cohort. **(C)** Clustering dendrograms of genes in ESCC. **(D)** Heatmap of the correlation analysis of module eigengenes with clinical phenotypes in ESCC. Red color represents positive correlation and blue color represents negative correlation. **(E)** Correlation between module membership and gene significance in blue modules. **(F)** Correlation between module membership and gene significance in black modules. **(G)** Venn diagram for intersecting genes between blue module in ESCC and the DEGs.

WGCNA was conducted on the ESCC dataset GSE29001 to systematically explore associations between gene expression patterns and clinical traits. Following network construction protocols, parameter optimization identified an optimal soft-thresholding power (β = 7) (Figures 2A,B). Based on the soft threshold of 7, we used the average linkage hierarchical clustering method to classify the TOM-based modules, each module contains no less than 60 genes. Modules with similarity greater than 75% were subsequently merged, and then the co-expression network architecture was resolved into 19 discrete gene clusters with distinct transcriptional coordination patterns (Figures 2C,D). We subsequently calculated the correlation of the traits and modules, discovering that the blue module most closely associated with tumor tissue (r = 0.91) and the black module exhibited the strongest association to normal tissue (r = 0.82) (Figure 2D). Furthermore, scatter plots show that a strong correlation was between module membership (MM) and gene significance (GS) (tumor cor = 0.88, normal tissue cor = 0.82) (Figures 2E,F). Ultimately, intersecting the blue module genes with DEGs revealed 1,019 overlapping candidates, suggesting their potential role in driving ESCC pathogenesis (Figure 2G).

### 3.3 Enrichment analysis of ESCC driver genes

**FIGURE 3:**
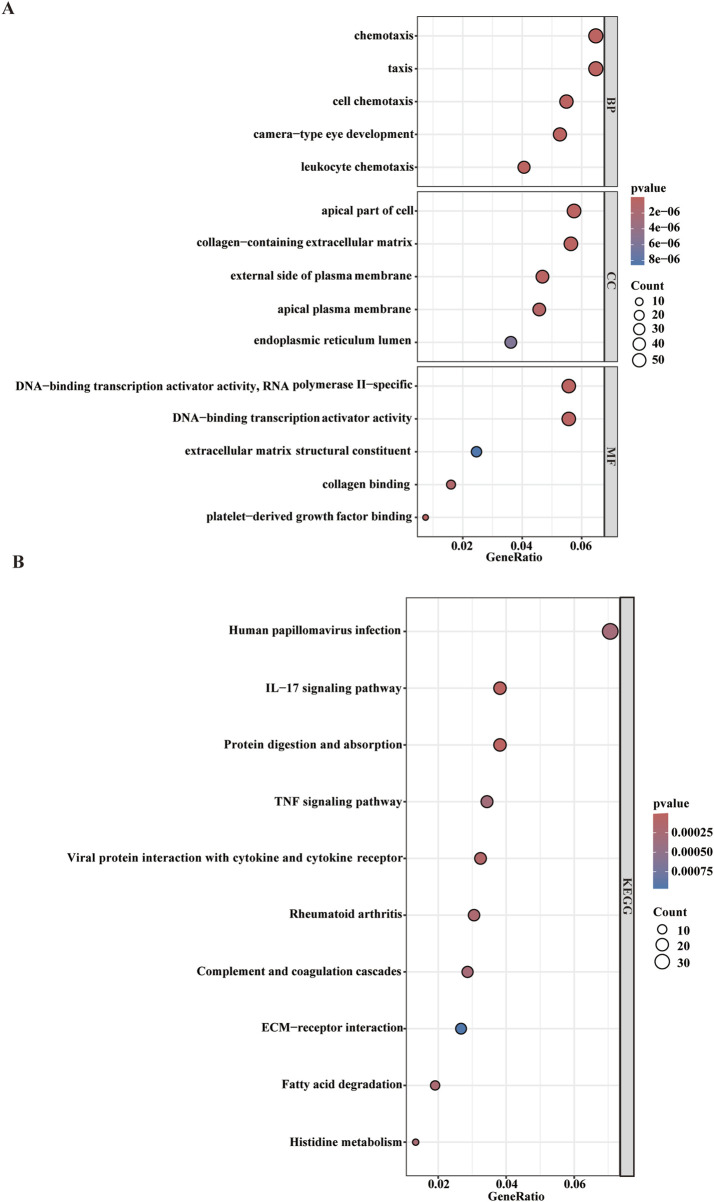
GO analysis and KEGG analysis. **(A)** GO analysis and **(B)** KEGG analysis of driver genes.

**FIGURE 4:**
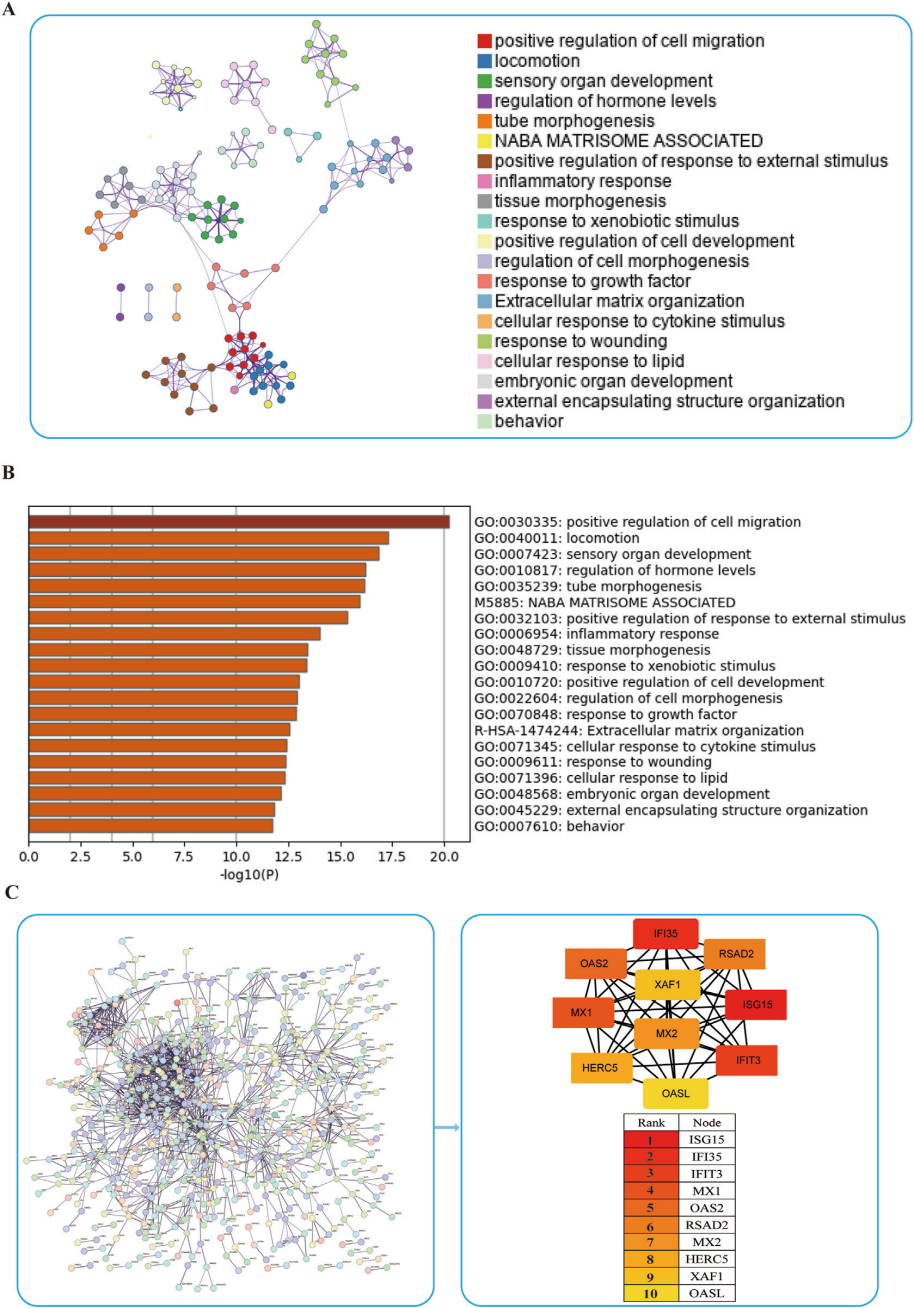
Enrichment of ESCC driver genes. **(A,B)** Enrichment analysis of 1,019 candidate driver genes using Metascape online tool. **(C)** PPI network analysis of driver genes.

While WGCNA-derived modules aggregate genes with analogous expression profiles, these genes might not encompass full spectrum of DEGs and may be quite different from DEGs that are critical for disease progression. To avoid omissions, we combined module genes and DEGs and identified 1,019 candidate driver genes. KEGG and GO were subsequently performed to elucidate the biological roles of these candidate genes. We found that the genes were primarily involved in pathways such as chemotaxis, taxis, and IL−17 signaling pathways (Figures 3A,B) Additionally, to further explore the enriched pathways associated with the marker genes, we discovered that different genes in the Metascape may show varying functional group distributions, with positive regulation of inflammatory responses being included (Figure 4A). Metascape-based enrichment analysis further highlighted that the positive regulation of inflammatory responses also contributes to the etiology of ESCC (Figure 4B). To classify genes into shared functional groups, the 1,019 candidate driver genes were analyzed using the String database, and non-interacting genes were systematically filtered out. Using the MCC algorithm in the Cytoscape software, we finally identified the top 10 genes in the PPI network from the mentioned genes (Figure 4C). Ultimately, *ISG15*, *IFI35, IFIT3*, *MX1*, *OAS2*, *RSAD2*, *MX2, HERC5*, *XAF1*, and *OASL* have been identified as potential diagnostic biomarkers.

### 3.4 Selection and validation of the hub genes with SVM and LASSO

**FIGURE 5:**
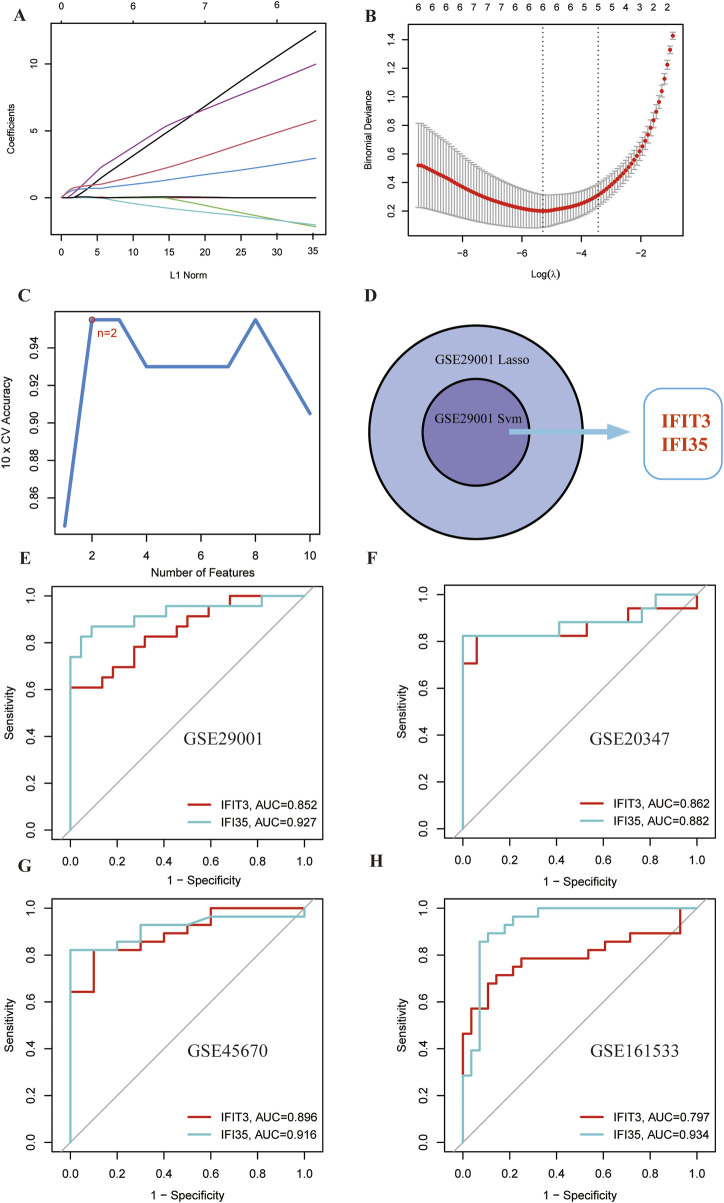
Selection and validation of shared hub genes by SVM and LASSO. **(A,B)** LASSO regression analysis of the GSE29001 cohort. **(C)** SVM analysis of the GSE29001 cohorts. **(D)** Cross-identification of optimal shared hub genes using SVM and LASSO. **(E)** ROC curves for two shared diagnostic markers in the GSE29001 cohort. **(F)** ROC curves for two shared diagnostic markers in the GSE20347 cohort. **(G)** ROC curves for two shared diagnostic markers in the GSE45670 cohort. **(H)** ROC curves for two shared diagnostic markers in the GSE161533 cohort.

In order to filter the most diagnostically valuable key genes, we utilized machine-learning algorithms to select the most significant features. Sequential SVM and LASSO regression analyses were conducted on the 10 candidate genes mentioned above. 6 genes were identified in the dataset by applying the LASSO method (Figures 5A,B). Concurrently, the SVM method filtered the 2 genes from the 10 genes (Figure 5C). The overlap of genes identified by different methods across various datasets ultimately pinpointed 2 common diagnostic biomarkers (*IFIT3* and *IFI35*) (Figure 5D).

The diagnostic potential of the hub genes was further validated via ROC curve analysis (Figure 5E). The AUC values for *IFIT3* (AUC = 0.852) and *IFI35* (AUC = 0.927) were both >0.7. It suggests that these two genes demonstrate strong diagnostic performance and could serve as potential biomarkers for ESCC.

**FIGURE 6:**
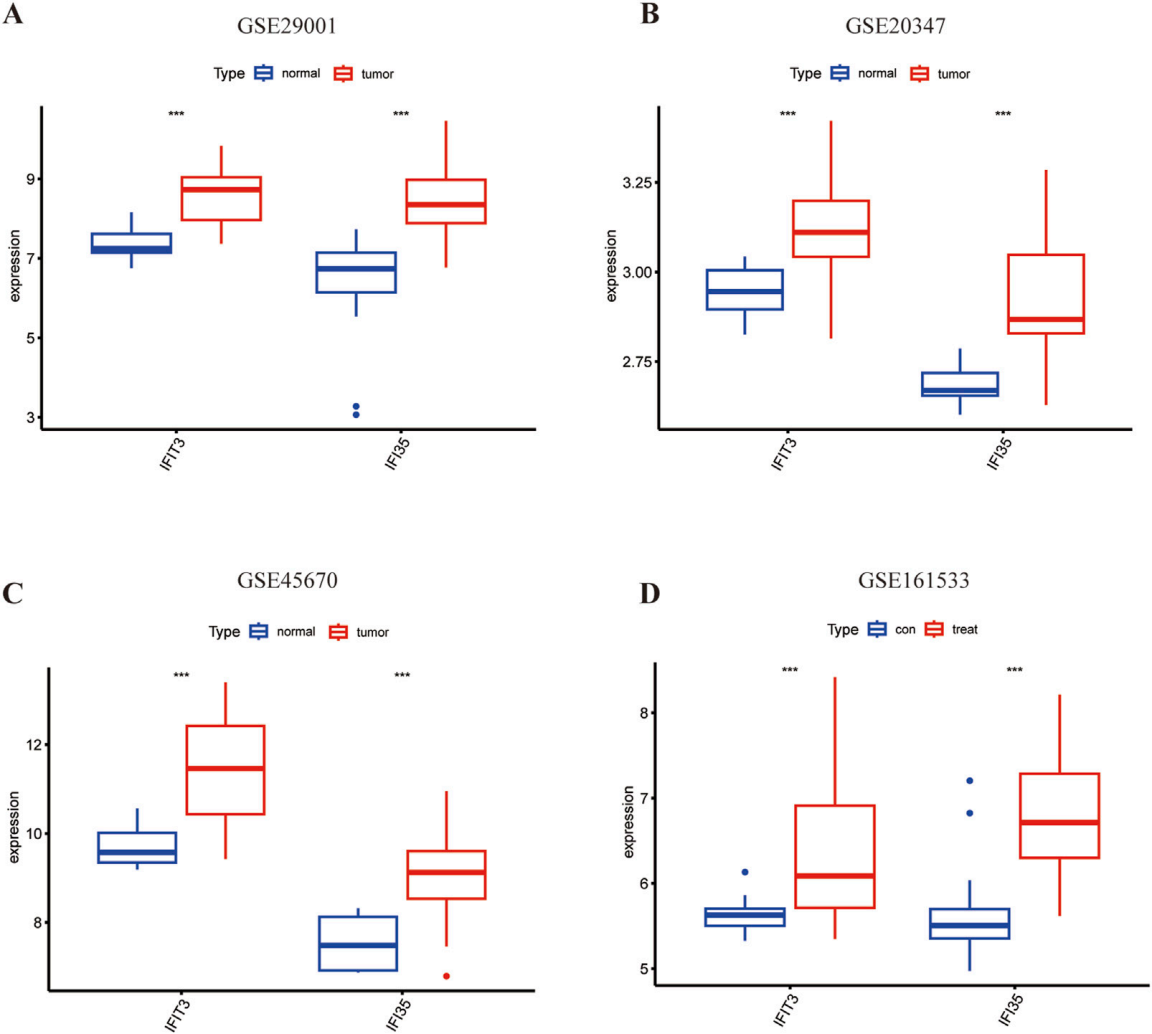
The expression of hub genes in ESCC. **(A)** Expression of two hub genes in GSE29001. **(B)** Expression of two hub genes in GSE20347. **(C)** Expression of two hub genes in GSE45670. **(D)** Expression of two hub genes in GSE161533. Blue color represents normal tissue and red color represents ESCC. *p < 0.05; **p < 0.01; ***p < 0.001; ****p < 0.0001.

The AUC values from the different cohorts also showed good predictive effectiveness in the validation sets (Figures 5F–H). The AUC values of *IFIT3* and *IFI35* in the validation set (GSE20347) were 0.862 and 0.882. In the validation sets (GSE45670 and GSE161533), the AUCs of *IFIT3* and *IFI35* were all above 0.700. The box plots revealed that both diagnostic markers were significantly upregulated in the disease group of the training set (Figure 6A). Crucially, consistent outcomes were observed across all validation sets (Figures 6B–D).

### 3.5 Immune cell infiltration and correlation with hub genes

**FIGURE 7:**
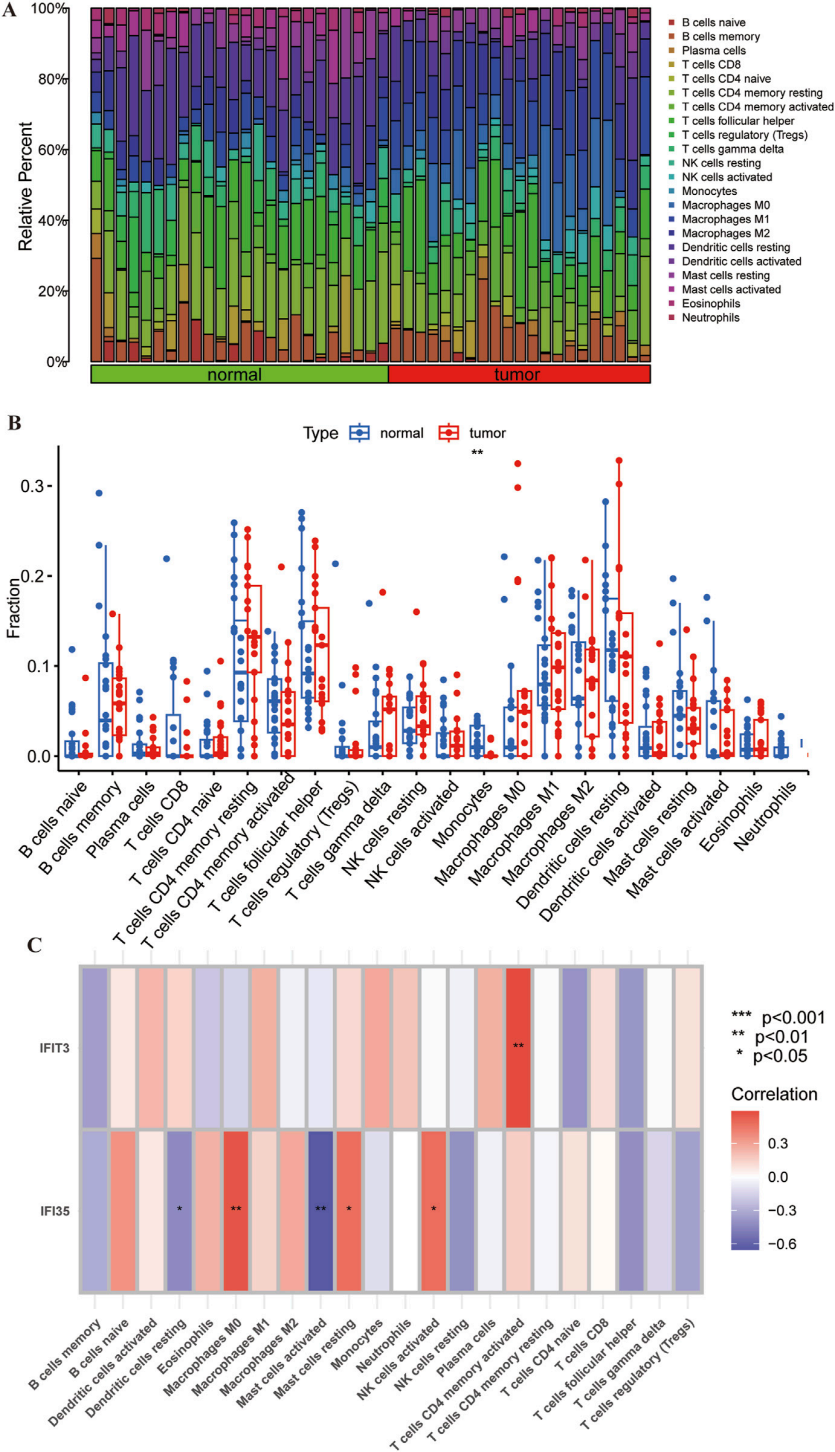
Correlation of immune cell infiltration and hub genes in ESCC. **(A)** Heatmap showing the differences in immune cell infiltration between the normal tissue and ESCC. **(B)** Boxplots showing the pattern of immune cell infiltration. **(C)** Heatmap showing the correlation between hub genes and immune cells. Red color represents positive correlation and purple color represents negative correlation. *p < 0.05; **p < 0.01; ***p < 0.001; ns, non-significant.

We investigated whether the CIBERSORT method could identify distinct immune infiltration patterns based on 22 immune cell types. The heatmap illustrates the differences in immune cell infiltration between normal tissue and tumor (Figure 7A). Differential expression analysis revealed a significant reduction of monocytes in tumor tissues compared to normal tissues, with statistical significance (Figure 7B).

Through supplementary analyses, we investigated the potential association of these two pivotal genes with immune cell infiltration within peripheral circulatory systems. Upon establishing significance, subsequent characterization revealed their demonstrated preferential correlations with specific immune cell subtypes. The correlation analysis revealed that *IFIT3* was positively correlated with T cells CD4 memory activated in the dataset. *IFI35* also showed a positive correlation with macrophages M0, mast cells resting, and activated NK cells activate, whereas negatively correlated with mast cells activated and dendritic cells resting (Figure 7C). It indicates that hub genes may regulate autoimmune responses by modulating immune cell expression.

### 3.6 Identification of potential drugs associated with hub genes

Based on analysis using the DSigDB library within Enrichr, 2 drugs (Tamibarotene and calcitriol) were identified through screening based on significant P-values after correction for multiple comparisons. The Odds Ratio values for both drugs are large, and although these numbers reflect enrichment of transcriptional features rather than binding affinity, these screened small molecules drugs can offer potential as therapeutic agents for ESCC (Table 1).

**TABLE 1:** Identification of potential drugs for ESCC based on the hub genes.

Term	P-value	Adjusted P-value	Odds ratio	Combined score	Overlap
Tamibarotene CTD 00002527	7.80E-04	0.021055	38882	278256.7424	2/559
Calcitriol CTD 00005558	0.00958	0.024973	36084	167721.6296	2/1958

## 4 Discussion

ESCC ranks among the most malignant cancers, being highly metastatic, and none of the therapeutic approaches yield good results (Reichenbach et al., 2019; Zhou et al., 2016; Yuan et al., 2022). Over the past decade, oncologists have increasingly turned their attention to targeted therapies, especially searching for a specific gene. A gene that strongly linked to the initiation and progression of ESCC could aid early diagnosis through level-specific testing. Additionally, targeting and suppressing its expression may provide a therapeutic strategy. Despite significant endeavors, achievements in this area have been minimal. Hence, exploring the regulatory pathways and critical targets of ESCC is vital for advancing early prevention and therapeutic strategies.

Initially, we conducted a differential analysis of GSE29001, identifying 4,122 DEGs, and applied WCGNA to identify modules with the highest correlation. By combining the WCGNA modules with DEGs, we identified 1,019 candidate driver genes. KEGG and GO enrichment analyses revealed that the chemotaxis and IL−17 signaling pathways were significantly upregulated. Research indicates that blocking chemotaxis not only suppresses tumor-induced osteomyelitis in metastatic castration-resistant prostate cancer (CRPC) patient subgroups, but also lowers circulating neutrophils and reduces intratumoral infiltration of CD11b+HLA-DRloCD15+CD14−myeloid cells. Additionally, it provides durable clinical advantages in metastatic CRPC subgroups via biochemical and radiographic responses (Guo et al., 2023). The IL−17-expressing CD4^+^ helper T cell (Th) subset is significantly involved in immune response signaling pathways, correlating not only with autoimmune diseases but also with cancer progression (Amatya et al., 2017). This indicated that these genes play a role in immune response regulation. Dysregulation of the immune system may be a primary contributor to ESCC development and progression. Further enrichment analysis using the Metascape database revealed a significant upregulation in the inflammatory response pathway, corroborating this view.

Next, utilizing 1,019 common driver genes, Within a systems biology framework, the Cytoscape platform was performed to generate a PPI network model, enabling systematic screening of topologically pivotal genes. Subsequent algorithmic curation identified a panel of 10 candidate genes exhibiting characteristics concordant with molecular signatures of diagnostic potential. For the refined selection of the diagnostic hub genes, LASSO and SVM analyses were utilized to pinpoint the optimal diagnostic biomarkers. Specifically, *IFIT3* and *IFI35* showed excellent diagnostic capabilities, validated by ROC curve analyses across the training and validation sets within the ESCC cohort. Notably, both genes showed a uniform upregulation trend in the tissue of ESCC relative to that in the normal tissue.

*IFIT3*, a member of the *IFIT* family with a four-peptide repeat sequence, is important in viral and immune system responses, and most studies have focused on antiviral and innate immunity (Platanias, 2005; Shi et al., 2019; Wu et al., 2022). Recent studies have highlighted the close association between *IFIT3* and tumors. Previous investigations utilizing bioinformatics approaches have identified elevated *IFIT3* expression in ESCC compared to adjacent normal tissues, a finding subsequently validated through analysis of clinical specimens. Furthermore, these studies demonstrated that patients exhibiting low *IFIT3* expression levels achieved significantly longer disease-free survival (DFS) and overall survival (OS) durations than those with high *IFIT3* expression (Cao et al., 2024). AND *IFIT3* may also contribute to chemotherapy resistance in pancreatic ductal adenocarcinoma, with transcriptomic analysis revealing enriched pathways in high *IFIT3* groups, such as inflammation, immune response, NF-κB signaling, and apoptosis (Wang et al., 2020). Moreover, *IFIT3* is overexpressed in head and neck squamous cell carcinoma, where it activates the PI3K/AKT pathway by targeting PD-L1, thereby promoting proliferation, migration, and invasion (Liu et al., 2024). *IFI35*, a 35 kDa interferon-induced protein, is broadly expressed in monocytes/macrophages, epithelial cells, and fibroblast. And it can regulate virus-associated immune-inflammatory responses across different cell types and tissues (Bange et al., 1994; Zheng et al., 2014; Das et al., 2014). *IFI35* showed completely opposite effects on tumors in different studies. A research indicates that *IFI35* promotes CD8^+^ T cell proliferation and cytotoxic activity through the PI3K/AKT/mTOR pathway in colorectal cancer, thus suppressing tumor growth (Li et al., 2023). In rectal cancer cell experiments, the upregulation of *IFI35* after X-ray exposure significantly suppressed CRC cell proliferation and colony formation. Additionally, G2 phase arrest along with increased production of reactive oxygen species (ROS), increased mitochondrial membrane potential, and elevated apoptosis rates were observed. Conversely, downregulation of *IFI35* caused the opposite effects. These findings indicate that *IFI35* upregulation significantly increases the radiosensitivity of CRC cells (Hu et al., 2021). These findings suggest that *IFI35* inhibits tumor progression, and in a separate study on triple-negative breast cancer, we observed that high expression of *IFI35* promotes CCL2 secretion, which leads to the infiltration of myeloid-derived suppressor cells (MDSC) and dysfunction of anti-tumor CD8^+^ T cells, thereby limiting its anti-tumor effects. In contrast, inhibition of *IFI35* expression improves immunotherapy outcomes (Xu et al., 2024). Studies on glioblastoma multiforme (GBM) have also demonstrated the tumor-promoting role of *IFI35*, suggesting that inhibiting the expression of it could offer a promising strategy for enhancing GBM treatment (Li et al., 2024). Our study, based on database analysis, found that *IFI35* expression is elevated in ESCC, and inhibiting its expression may help suppress the tumorigenesis and progression of ESCC. In summary, both hub genes (*IFIT3* and *IFI35*) are involved in immune responses and could be potential biomarkers for ESCC.

In summary, it is evident that immune responses are critically involved in the enriched pathways of differential genes and the tumor-related mechanisms of hub genes. We subsequently applied CIBERSORT to examine the immune cell infiltration in ESCC. Monocyte infiltration was markedly diminished in ESCC tissues relative to adjacent normal tissues, as evidenced by comparative analysis. Human monocytes are categorized into three distinct subsets: classical/inflammatory, non-classical/surveillance, and intermediate subtype (Ugel et al., 2021). Recent study indicates that in the process of tumor development, different monocyte populations play divergent roles, where certain subsets facilitate tumor growth whereas others suppress oncogenesis. Similar dynamics are observed during tumor metastasis (Chen et al., 2023). Thus, the function of monocytes and their lineage-derived cells in ESCC tumorigenesis and therapy necessitate more profound mechanistic exploration. Analysis of key genes and immune infiltration in peripheral blood revealed that *IFIT3* positively correlates with T cell CD4 memory activated, while *IFI35* is positively associated with Mast cells resting, NK cells activated, and Macrophages M0, but negatively correlated with Mast cells activated and Dendritic cells. These differences in immune cell expression may significantly affect the prognosis of ESCC.

By mining datasets from the GEO database, this study screened and validated the upregulated genes *IFIT3* and *IFI35* in ESCC, subsequently performing functional enrichment analysis, infiltration investigation, and drug prediction. Building upon the aforementioned investigations, we have identified both IFIT3 and IFI35 as promising candidate therapeutic targets in ESCC. However, this study also has limitations. The study in this paper is only limited to the analysis of data, and there is a lack of *in vivo* and *in vitro* experiments for further research. Further functional and pathway studies are warranted. We plan to address these issues in the future to better present our point of view.

Our research investigated the hub genes of ESCC, focusing on two critical genes, *IFIT3* and *IFI35,* which have been validated in various datasets. These findings indicate their potential as biomarkers of ESCC.

## Data Availability

Publicly available datasets were analyzed in this study. This data can be found here: https://www.ncbi.nlm.nih.gov/geo/.
