# Supplementary material for: IFI35 and IFIT3 are potentially important biomarkers for early diagnosis and treatment of esophageal squamous cell carcinoma: based on WGCNA and machine learning analysis
Source: Front Genet. 2025 May 20;16:1583202. doi: 10.3389/fgene.2025.1583202 (PMC12129983; doi:10.3389/fgene.2025.1583202)
Supplement: Supplementary file 1 [file DataSheet1.zip › Raw Data/05ppi/metsscape/Enrichment_GO_ColorByCluster.pdf]

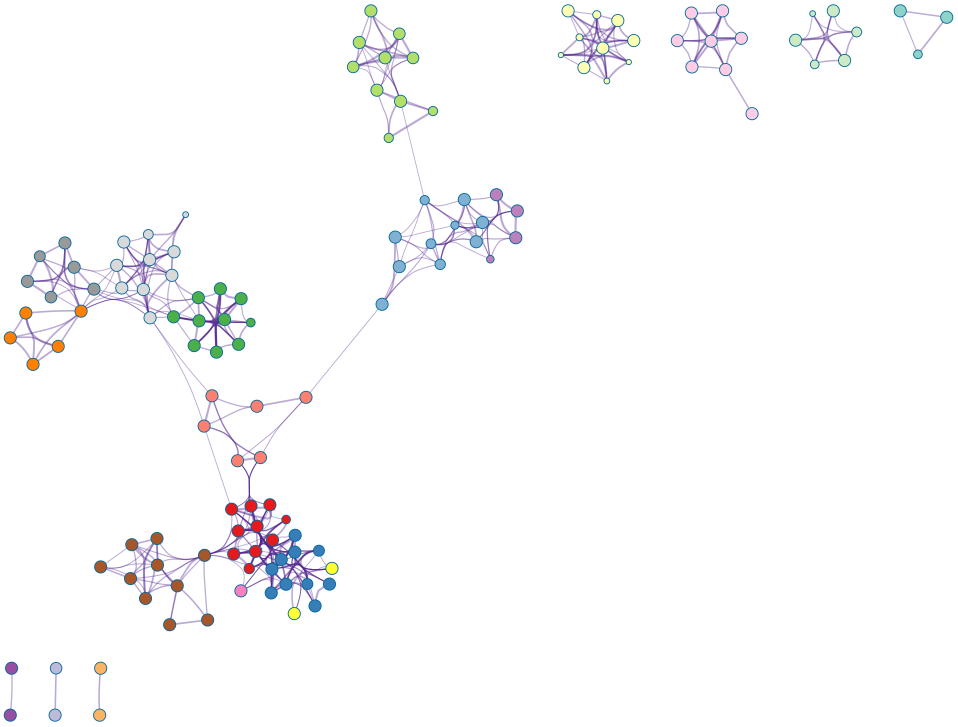

- positive regulation of cell migration
- locomotion
- sensory organ development
- regulation of hormone levels
- tube morphogenesis
- NABA MATRISOME ASSOCIATED
- positive regulation of response to external stimulus
- inflammatory response
- tissue morphogenesis
- response to xenobiotic stimulus
- positive regulation of cell development
- regulation of cell morphogenesis
- response to growth factor
- Extracellular matrix organization
- cellular response to cytokine stimulus
- response to wounding
- cellular response to lipid
- embryonic organ development
- external encapsulating structure organization
- behavior

created by
http://metascape.org
