# Supplementary figures and images for: IFI35 and IFIT3 are potentially important biomarkers for early diagnosis and treatment of esophageal squamous cell carcinoma: based on WGCNA and machine learning analysis
Source: Front Genet. 2025 May 20;16:1583202. doi: 10.3389/fgene.2025.1583202 (PMC12129983; doi:10.3389/fgene.2025.1583202)

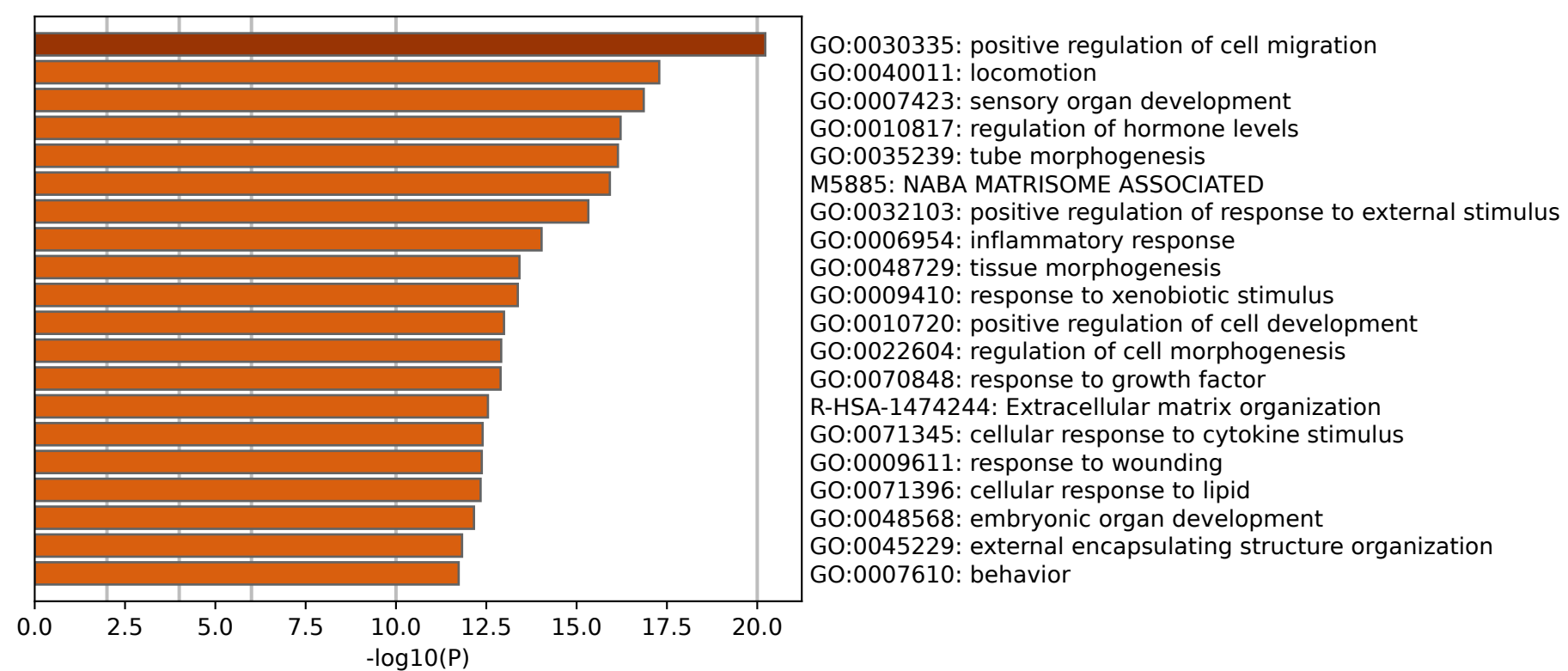

Supplement: Supplementary file 1 [file DataSheet1.zip › Raw Data/05ppi/metsscape/Enrichment_heatmap_HeatmapSelectedGO.pdf]

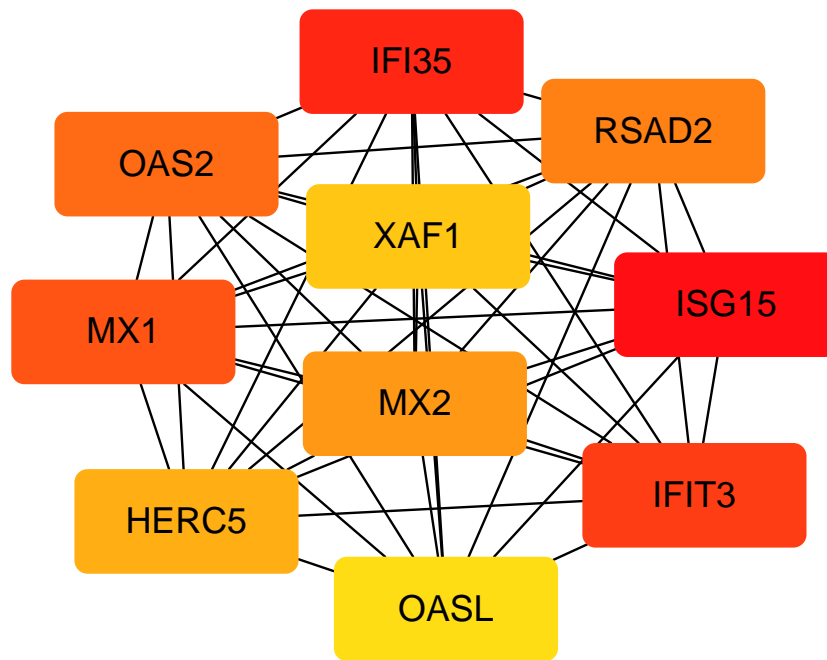

Supplement: Supplementary file 1 [file DataSheet1.zip › Raw Data/05ppi/string/string_interactions_short.tsv_MCC_top10.pdf]

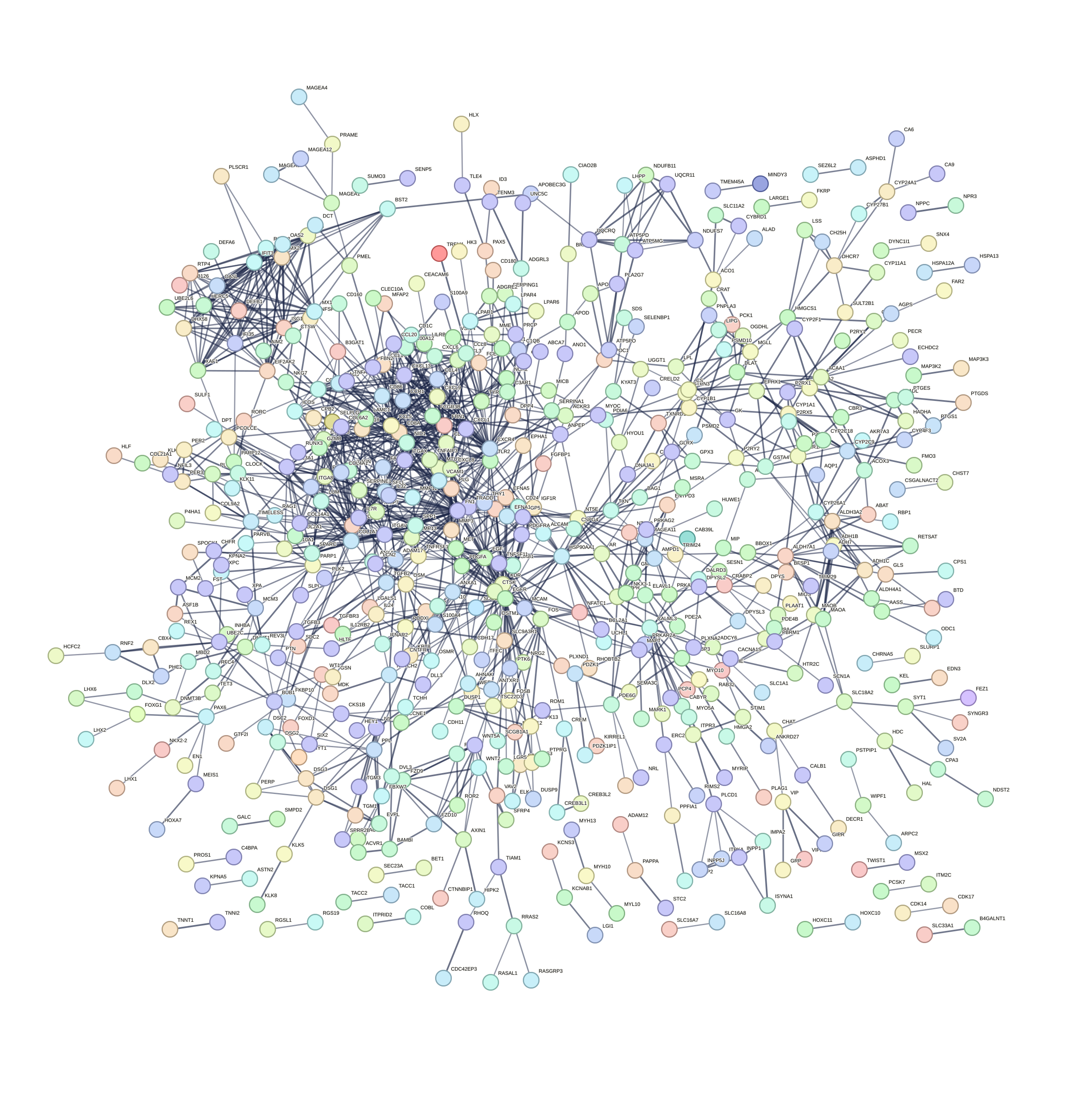

Supplement: Supplementary file 1 [file DataSheet1.zip › Raw Data/05ppi/string/string_normal_image.png]

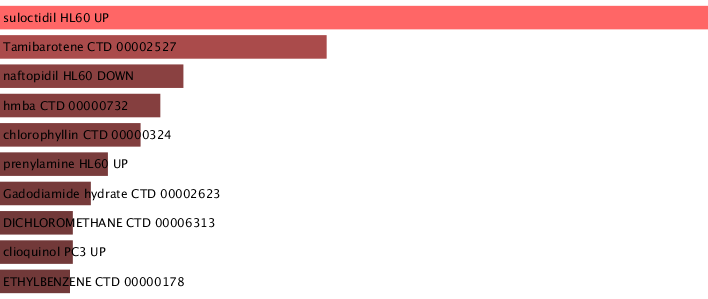

Supplement: Supplementary file 1 [file DataSheet1.zip › Raw Data/09Drug/DSigDB_bar_graph.png]
